# Magnetic Poly(N-isopropylacrylamide) Nanocomposites: Effect of Preparation Method on Antibacterial Properties

**DOI:** 10.1186/s11671-017-2341-0

**Published:** 2017-10-19

**Authors:** Nhung H. A. Nguyen, Mohamed S. A. Darwish, Ivan Stibor, Pavel Kejzlar, Alena Ševců

**Affiliations:** 1 Institute for Nanomaterials, Advanced Technologies and Innovation, Technical University of Liberec, 461 17 Liberec, Czech Republic; 2 Faculty of Mechatronics, Informatics and Interdisciplinary Studies, Technical University of Liberec, 461 17 Liberec, Czech Republic; 3 Egyptian Petroleum Research Institute, 1 Ahmed El-Zomor Street, El Zohour Region, Nasr City, Cairo 11727 Egypt

**Keywords:** Magnetic poly(N-isopropylacrylamide), PNIPAAm, Bio-application, *Escherichia coli*, *Staphylococcus aureus*

## Abstract

The most challenging task in the preparation of magnetic poly(N-isopropylacrylamide) (Fe_3_O_4_-PNIPAAm) nanocomposites for bio-applications is to maximise their reactivity and stability. Emulsion polymerisation, in situ precipitation and physical addition were used to produce Fe_3_O_4_-PNIPAAm-1, Fe_3_O_4_-PNIPAAm-2 and Fe_3_O_4_-PNIPAAm-3, respectively. Their properties were characterised using scanning electron microscopy (morphology), zeta-potential (surface charge), thermogravimetric analysis (stability), vibrating sample magnetometry (magnetisation) and dynamic light scattering. Moreover, we investigated the antibacterial effect of each nanocomposite against Gram-negative *Escherichia coli* and Gram-positive *Staphylococcus aureus*. Both Fe_3_O_4_-PNIPAAm-1 and Fe_3_O_4_-PNIPAAm-2 nanocomposites displayed high thermal stability, zeta potential and magnetisation values, suggesting stable colloidal systems. Overall, the presence of Fe_3_O_4_-PNIPAAm nanocomposites, even at lower concentrations, caused significant damage to both *E. coli* and *S. aureus* DNA and led to a decrease in cell viability. Fe_3_O_4_-PNIPAAm-1 displayed a stronger antimicrobial effect against both bacterial strains than Fe_3_O_4_-PNIPAAm-2 and Fe_3_O_4_-PNIPAAm-3. *Staphylococcus aureus* was more sensitive than *E. coli* to all three magnetic PNIPAAm nanocomposites.

## Background

Magnetic thermoresponsive polymer nanocomposites have been used for a wide range of applications, including water treatment and nanomedicine [1–4]. Each nanocomposite is specifically designed to benefit from the combination of features inherent in both components, i.e. magnetic particles and temperature-responsive polymers, thus creating a nanocomposite that is more specific and controllable. Magnetite (Fe_3_O_4_) nanoparticles impart magnetic properties that allow for rapid and easy separation following application of an external magnetic field [5]. Poly(N-isopropylacrylamide) (PNIPAAm) forms a three-dimensional hydrogel that undergoes a reversible lower critical solution temperature (LCST) phase transition from a single coil with a swollen hydrated state to a collapsed and shrunken dehydrated state [6] when heated in water above 32 °C. Capping of the magnetic nanoparticles with a PNIPAAm layer not only provides colloidal stability in water but also allows for surface functionality by binding with other molecules, such as drugs, proteins or enzymes [7]. Construction of dual responsive nanocomposites is achieved by combining two properties that respond simultaneously to a combination of temperature and magnetism. The most common methods used for synthesis of Fe_3_O_4_-PNIPAAm nanocomposites are physical addition, in situ precipitation and emulsion polymerisation. Physical addition, the simplest method, requires the physical mixing of previously synthesised magnetic nanoparticles and PNIPAAm particles. The second method, in situ precipitation, involves precipitation of magnetic nanoparticles in the presence of the PNIPAAm nanopolymer [8]. The third (and most common) route, emulsion polymerisation, requires polymerisation of the (N-isopropylacrylamide) monomer in the presence of magnetic nanoparticles [9–11]. Fe_3_O_4_-PNIPAAm nanocomposites have found widespread use in biomedical and biotechnological applications. Highly stable, controlled and well-dispersed magnetic nanoparticles will be required in order to increase the suitability of such nanocomposites for future applications. One recent innovation involves an external magnetic field that creates a local heat source for self-heating particles, causing the PNIPAAm to shrink and in turn allowing release of encapsulated drugs [12]. This phenomenon, coupled with magnetic beads targeted on tumours, opens up other potential cancer therapies such as hyperthermia. Hyperthermia can be initiated by oscillating nanoparticles in an oscillating magnetic field at frequencies ranging from kilohertz to megahertz. Other Fe_3_O_4_-PNIPAAm nanocomposites have recently been synthesised to control the release of bio-active molecules, such as myoglobin or vitamin B12, and for drug delivery [13]. A recent study using PNIPAAm-coated superparamagnetic Fe_3_O_4_ nanoparticles was able to show that thermally induced aggregation of iron oxide nanoparticles greatly increases T2 contrast during magnetic resonance imaging [14]. Clearly, Fe_3_O_4_-PNIPAAm shows great promise for future developments in both biomedical and biotechnological applications. Consequently, it is important that further studies are undertaken on the biocompatibility of this material and its antibacterial effect.

In this study, we investigated the effect of three preparation methods on the physical-chemical properties of Fe_3_O_4_-PNIPAAm nanocomposites. In doing so, we aim to assess the most convenient preparation method for producing nanocomposites displaying enhanced properties for biological applications. For the first time, we also describe the antibacterial effects of the three Fe_3_O_4_-PNIPAAm nanocomposites using a multi-endpoint approach, bacterial growth rate, viability, cell morphology and level of DNA damage.

## Methods

### Chemicals

Iron(III) chloride hexahydrate (FeCl_3_.6H_2_O, ≥ 98%), Iron(II) chloride tetrahydrate (FeCl_2_.4H_2_O, ≥ 99%), ammonium hydroxide (26% NH_3_ in H_2_O), N-isopropyl-acrylamide (NiPAM, ≥ 99%), N,N-methylenebis(acrylamide) (BIS, ≥ 99%), sodium dodecyl sulphate (SDS, ≥ 99%) and ammonium persulphate (APS, ≥ 98.5%) were all purchased fresh from Sigma-Aldrich, Germany.

### Preparation of PNIPAAm by Emulsion Polymerisation

NiPAM (4 g), BIS (0.2 g) and SDS (0.3 g) were dissolved in 350 ml of deionised water (DI) at 70 °C under atmospheric nitrogen. APS (0.0035 g) was dissolved in 1 ml of DI and added to the reaction vessel to start the reaction. After 4 h, the reaction was stopped and the prepared particles washed with DI water. Finally, the PNIPAAm nanoparticles were separated by centrifugation (12,000 rpm for 30 min) and used in further reactions.

### Preparation of Magnetite (Fe_3_O_4_) Nanoparticles

FeCl_2_·4H_2_O (1.9 g) and FeCl_3_·6H_2_O (5.4 g) (molar ratio 1:2) were dissolved in DI (100 ml) and heated to 70 °C. Ammonium hydroxide (NH_4_OH; 6 ml) was quickly added to the solution, which immediately produced a deep black magnetic precipitate. Finally, the Fe_3_O_4_ nanoparticle suspension was stirred for 30 min at 70 °C. The product was washed several times with DI, following which the Fe_3_O_4_ nanoparticles were dried in a rotary evaporator (25 mbar at 40 °C) until a fine powder was formed. This was used in all further reactions.

### Preparation of Magnetic PNIPAAm Nanocomposite by Emulsion Polymerisation (Fe_3_O_4_-PNIPAAm-1)

NiPAM (0.4 g), freshly prepared Fe_3_O_4_ nanoparticles (0.2 g), BIS (0.2 g) and SDS (0.3 g) were dissolved in 350 ml of DI and heated to 70 °C under a nitrogen atmosphere. APS (0.0035 g) was then dissolved in 1 ml of DI and added to the reaction vessel to start the reaction. After 4 h, the reaction was stopped and the prepared nanocomposite washed with DI. Finally, Fe_3_O_4_-PNIPAAm-1 was separated out by centrifugation (12,000 rpm for 30 min) and then dried using a rotary evaporator (25 mbar at 40 °C). The powdered material was stored in the dark at room temperature.

### Preparation of Magnetic PNIPAAm Nanocomposite Through In Situ Precipitation (Fe_3_O_4_-PNIPAAm-2)

FeCl_2_ (0.148 g), FeCl_3_ (0.4 g) and 10 ml DI were mixed well and added to 1 g of PNIPAAm. NH_4_OH (3 ml) was then quickly added to the solution, which immediately produced a deep black magnetic precipitate. The suspension was then stirred for 30 min at 70 °C. The prepared nanocomposite was washed with DI, following which the Fe_3_O_4_-PNIPAAm-2 was separated out by centrifugation (12,000 rpm for 30 min) and dried using a rotary evaporator (25 mbar at 40 °C). The resultant powder was stored in the dark at room temperature.

### Preparation of Magnetic PNIPAAm Nanocomposite Through Physical Addition (Fe_3_O_4_-PNIPAAm-3)

Freshly prepared PNIPAAm (1 g), freshly prepared Fe_3_O_4_ nanoparticles (0.5 g) and DI (5 ml) were mixed well, and the resultant suspension stirred for 30 min at 70 °C. The nanocomposite thus prepared was washed with DI, following which the Fe_3_O_4_-PNIPAAm-3 was separated out through centrifugation (12,000 rpm for 30 min) and dried using a rotary evaporator (25 mbar at 40 °C). The powdered material was stored in the dark at room temperature.

### Nanocomposites Characterisation

The size and zeta potential of the Fe_3_O_4_-PNIPAAm nanocomposites were measured following complete dissolution of the nanoparticles in DI (dispersal in DI followed by sonification for 2 min at room temperature). Zeta potential measurements were performed using a Zetasizer Nano analyser (Malvern Instruments, USA) at pH 7. A Zetasizer Nano dynamic light scattering (DLS) unit was employed to measure the hydrodynamic diameter of particle aggregates in DI. Thermogravimetric analysis (TGA) was undertaken in order to quantify the amount of coating and to determine the nanocomposite’s thermal stability. Thermal studies were undertaken on 3–4 mg of dry sample at temperatures ranging from 25 to 900 °C, using a TGA Q500 (TA Instruments, USA) under a nitrogen atmosphere (heating rate 10 °C/min). The material’s magnetic properties were measured using a MicroMag™ 2900 vibrating sample magnetometer (Princeton measurements corporation, USA). Microscopy images were obtained using a scanning electron microscope (SEM), the particles being first thoroughly dissolved in DI and a drop of the solution placed on the copper grid of a Zeiss ULTRA Plus field-emission SEM equipped with a Schottky cathode. All images were analysed using Smart SEM software v 5.05 (Zeiss, Germany) for imaging operated at 1.5 kV.

### Bacterial Strains and Media

Gram-negative *Escherichia coli* CCM3954 and Gram-positive *Staphylococcus aureus* CCM 3953 (Brno, Czech Republic) were used for all experiments. Detailed information on the strains is provided on the web page of the ‘Czech Collection of Microorganisms’ (http://www.sci.muni.cz/ccm/). Each bacterial culture was freshly prepared and held overnight in a soya nutrient broth (Sigma-Aldrich) before performing the biological experiments.

### DNA Damage

Comet assays were performed following the methodology of Singh et al. [15] and Solanky et al. [16]. All chemicals were purchased from PENTA (Czech Republic) unless otherwise noted. A fresh bacterial culture (adjusted to 10^7^ cells/ml) was grown overnight and then incubated with two concentrations (0.1 and 1 g/l) of PNIPAAm and each of the Fe_3_O_4_-PNIPAAm nanocomposites for 30 min at 37 °C.

A microgel was prepared by putting 100 ml of agarose onto the frosted surface of a slide and covering it with a 24 × 50 mm cover glass (ThermoFisher Scientific, USA). The slides were left at room temperature for 5 min, then the cover glasses were removed and the slides allowed to dry. This dried agarose layer (first layer) provided a firm base for subsequent layers. After exposing the bacteria to the PNIPAAm and Fe_3_O_4_-PNIPAAm nanocomposites for 30 min, 2 μl (containing approximately 10,000 exposed cells) was taken and mixed with 100 μl of freshly prepared 0.5% agarose. This mixture was pipetted onto frosted slides and immediately covered with a cover glass (second layer). The slides were then cooled in a steel tray over ice. The cover glasses were removed after 1 min, and a third layer of 100 μl of lysis agarose (including 0.5% agarose with 5 μg/ml RNAse A [Ameresco, USA], 0.25% sodium N-lauroylsarcosine and 0.5 mg/ml lysozyme) was produced, again using a cover glass. The slides were then left on ice for 10 min then placed into a humid chamber for 30 min at 37 °C. After removing the cover glass, the slides were immersed in a lysing solution containing 2.5 M of NaCl, 100 mM of EDTA tetrasodium salt, 10 mM tris buffer of pH 10, 1% sodium lauroyl sarcosine and 1% triton X-100. After 1 h of lysis at room temperature, the slides were transferred to an enzyme digestion solution containing 2.5 M of NaCl, 10 mM of EDTA and 10 mM tris pH 7. Four buffer with 1 mg/ml of proteinase K. The slides were then incubated at 37 °C for 2 h, following which they were placed on the horizontal slab of an electrophoretic unit (Scie-plas, UK) and equilibrated with 300 mM of sodium acetate and 100 mM pH 9 tris buffer for 20 min then electrophoresed at 12 V (0.4 V/cm, approximately 100 mA) for 30 min. Following electrophoresis, the slides were immersed in 1 M ammonium acetate in ethanol (5 ml of 10 M ammonium acetate and 45 ml of absolute ethanol) for 20 min, absolute ethanol for 0.5 h and 70% ethanol for 10 min, after which the slides were air-dried at room temperature. To achieve uniform staining, the slides were pretreated with 50 ml of a freshly prepared solution of 5% TE buffer and 10 mM of NaH_2_PO_4_. The slides were then stained with 50 μl of a freshly prepared 1 mM solution of SYBR stain (Sigma-Aldrich, USA) in TE buffer for 30 min. Migration of DNA strand breaks (comets) was visualised using an AxioImager fluorescence microscope at × 400 magnification and AxioVision v 4 software (Zeiss, Germany). Typically, a tail length of 50 comets was individually measured for each sample.

### Bacterial Growth Rate, Cell Viability and Morphology

The experimental protocol followed that described in Darwish et al. [17]. Briefly, a Fe_3_O_4_-PNIPAAm nanocomposite stock suspension (10 g/l) was added to fresh bacterial culture in order to obtain final concentrations of 0.01, 0.05, 0.5 and 1 g/l. Each concentration was produced in triplicate in a 24-well plate. Negative controls, consisting of bacterial cells only in growth media and Fe_3_O_4_-PNIPAAm nanocomposite only in growth media, were run in parallel. The plate was then incubated at 37 °C, following which the sample’s optical density was measured at 600 nm (OD600) every 2 h for 6 h using a Synergy™ HTX plate reader (Biotek, USA). Bacterial growth rate was defined as the R linear regression of the OD600 measurement (absorbance units, AU) versus incubation time in hours. Preliminary measurements of nanocomposite samples without cells (6 h at 600 nm) showed constant absorbance values that did not interfere with absorbance values of nanocomposites measured with bacterial cells.

The effective concentration of nanocomposite at 10% inhibition (EC10) on bacterial growth rate (*µ*) was calculated for each form of Fe_3_O_4_-PNIPAAm based on the equation: *I*(%) = (*µ*_*C*_−*µ*_*T*_)/*µ*_*C*_×100, where *I* is inhibition, *µ*_*C*_ is the mean control growth rate value and *µ*_*T*_ is the growth rate of the culture affected by the nanocomposite [18].

After 24-h incubation, 100 μl aliquots of each sample were stained using the L7007 Bacterial Viability Kit (Molecular Probes, Invitrogen, USA) in the dark for 15 min. Determination of the proportion of live (Ex/Em 485/528 nm) and dead cells (Ex/Em 485/645 nm) was performed using a Synergy™ HTX plate reader (Biotek, USA). The percentage of dead cells was calculated as the ratio of dead to live cells. At the same time, images of *E. coli* and *S. aureus* were obtained using an AxioImager fluorescence microscope (Zeiss, Germany) with Ex/Em 470/490–700 nm. The length of *E. coli* cells and area of *S. aureus* cell clusters were determined at × 600 magnification using AxioVision v 4 software (Zeiss, Germany).

### Statistical Analysis

Differences between bacterial strains incubated in PNIPAAm, different Fe_3_O_4_-PNIPAAm nanocomposites and control samples without nanocomposites were tested using ANOVA and Dunnett’s test (GraphPad PRISM, USA).

## Results

**Fig. 1:**
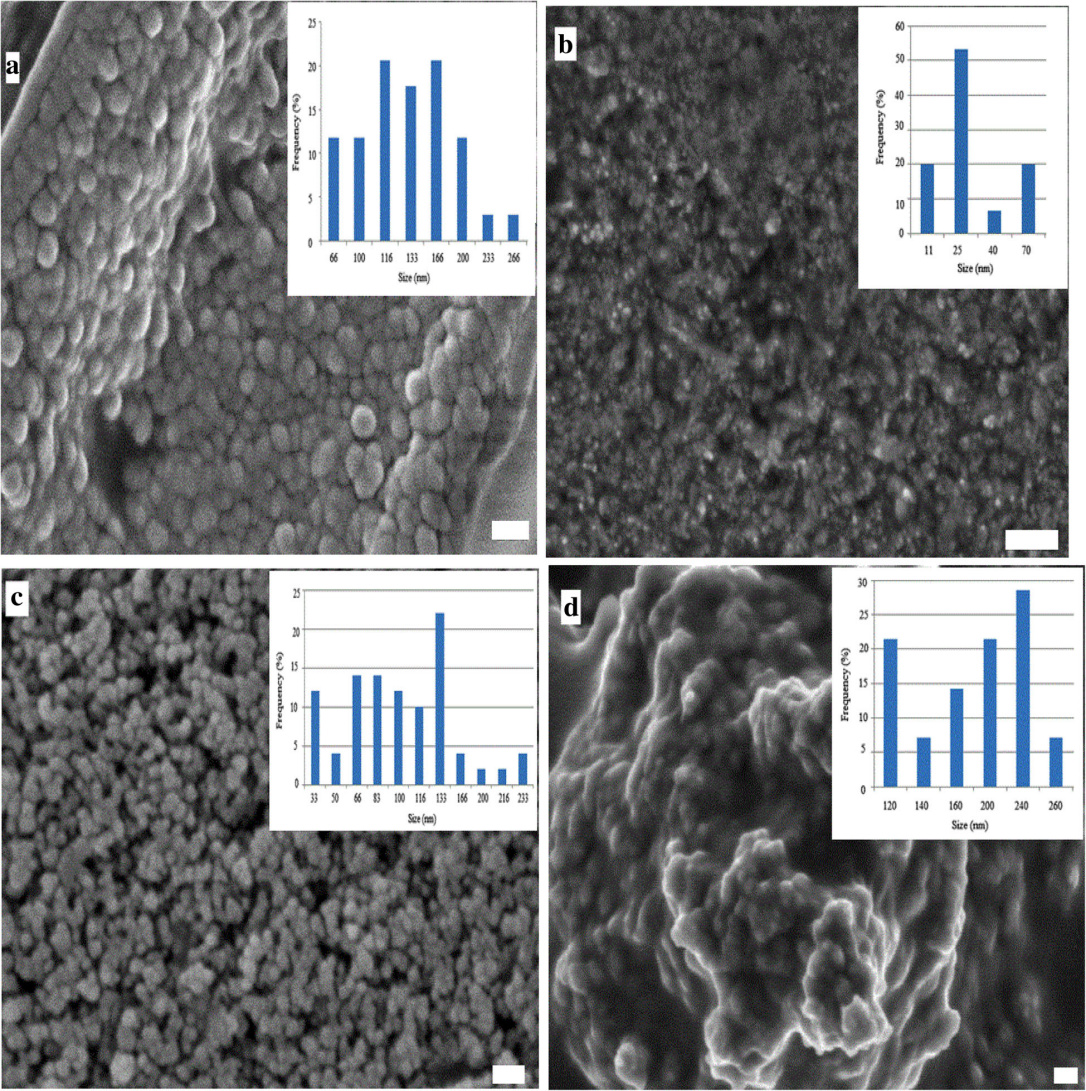
Scanning electron microscope images and histograms of PNIPAAm (**a**), Fe_3_O_4_-PNIPAAm-1 (**b**), Fe_3_O_4_-PNIPAAm-2 (**c**) and Fe_3_O_4_-PNIPAAm-3 (**d**). Scale bar = 200 nm

In this study, we synthesised Fe_3_O_4_-PNIPAAm nanocomposite employing three different protocols: emulsion polymerisation (Fe_3_O_4_-PNIPAAm-1), in situ precipitation (Fe_3_O_4_-PNIPAAm-2) and physical addition (Fe_3_O_4_-PNIPAAm-3). SEM imaging showed that the type of protocol used had a clear effect on sample morphology and particle size, with Fe_3_O_4_-PNIPAAm-1, Fe_3_O_4_-PNIPAAm-2 and Fe_3_O_4_-PNIPAAm-3 showing a broad size distribution, agglomeration due to high surface energy between nanoparticles and presence of magnetic dipolar interactions **(**Fig. 1 **)**.

**Fig. 2:**
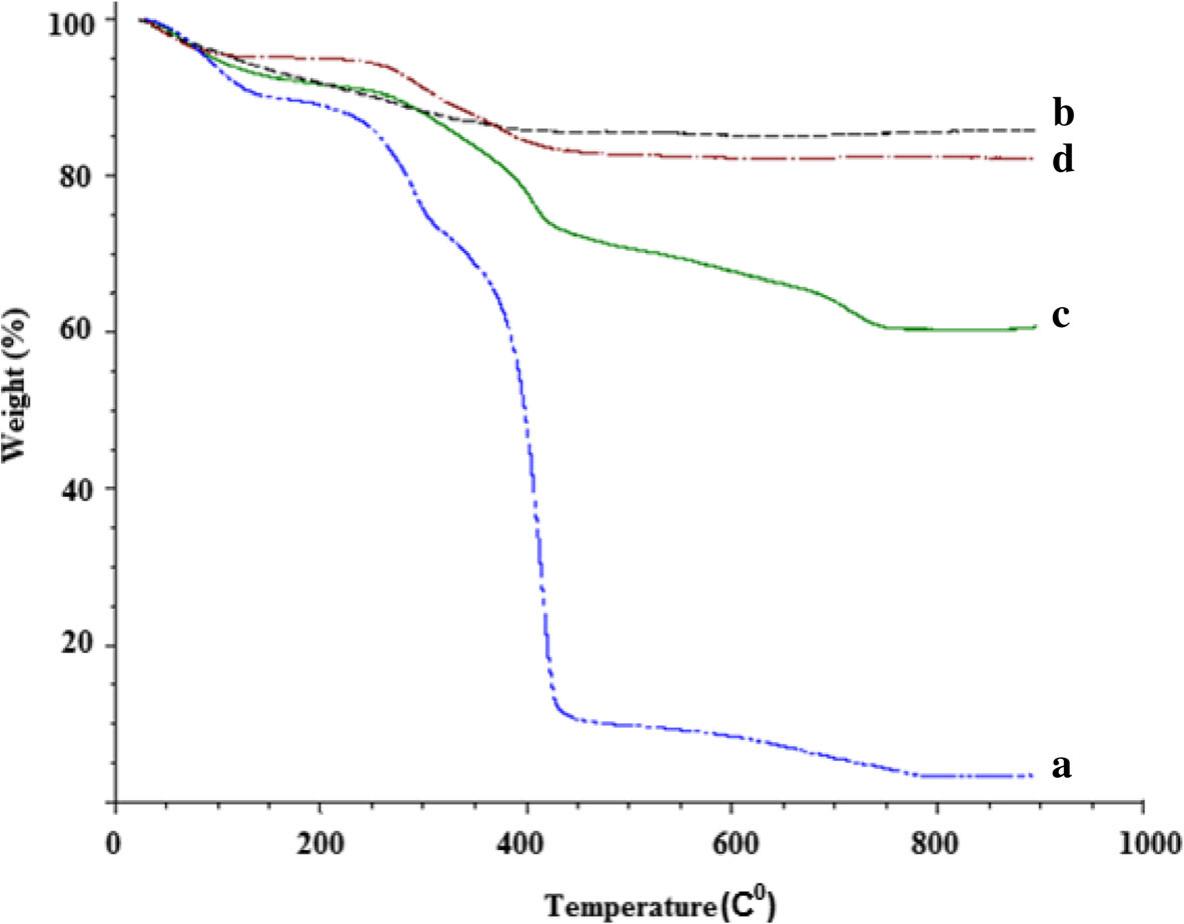
Thermogravimetric analysis of PNIPAAm (a), Fe_3_O_4_-PNIPAAm-1 (b), Fe_3_O_4_-PNIPAAm-2 (c) and Fe_3_O_4_-PNIPAAm-3 (d)

**Fig. 3:**
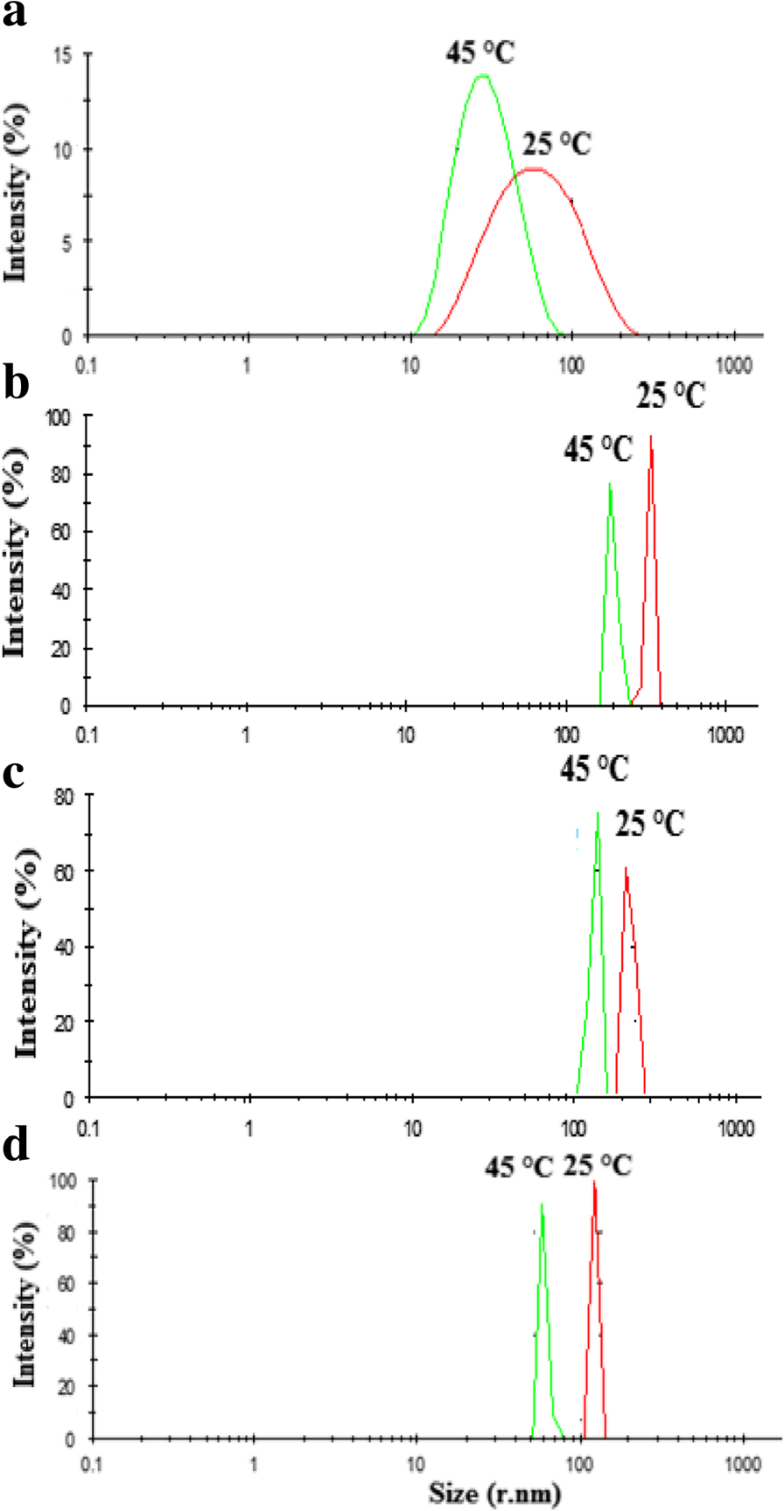
Dynamic light scattering below (25 °C) and above (45 °C) the lower critical solution temperature phase transition for PNIPAAm (**a**), Fe_3_O_4_-PNIPAAm-1 (**b**), Fe_3_O_4_-PNIPAAm-2 (**c**) and Fe_3_O_4_-PNIPAAm-3 (**d**)

TGA indicated that the Fe_3_O_4_-PNIPAAm samples became relatively stable at temperatures above 400 °C (Fig. 2). Overall, PNIPAAm nanoparticles showed lower residual content than the Fe_3_O_4_-PNIPAAm nanocomposites. Zeta potential values for surface charge were − 1.58 mV for PNIPAAm, − 15.6 mV for Fe_3_O_4_-PNIPAAm-1, − 16.4 mV for Fe_3_O_4_-PNIPAAm-2 and − 1.8 mV for Fe_3_O_4_-PNIPAAm-3. Vibrating sample magnetometer values for magnetisation saturation were 50.4 emu/g for Fe_3_O_4_-PNIPAAm-1, 53.7 emu/g for Fe_3_O_4_-PNIPAAm-2 and 21.0 emu/g for Fe_3_O_4_-PNIPAAm-3. Dynamic light scattering above (45 °C) and below (25 °C) LCST indicated a hydrodynamic size for PNIPAAm of 50 nm at 25 °C and 27 nm at 45 °C; 412 nm at 25 °C and 197 nm at 45 °C for Fe_3_O_4_-PNIPAAm-1; 212 nm at 25 °C and 130 nm at 45 °C for Fe_3_O_4_-PNIPAAm-2 and 122 nm at 25 °C and 60 nm at 45 °C for Fe_3_O_4_-PNIPAAm-3 (Fig. 3).

### Fe_3_O_4_-PNIPAAm Nanocomposite Effect on the Bacterial DNA

**Fig. 4:**
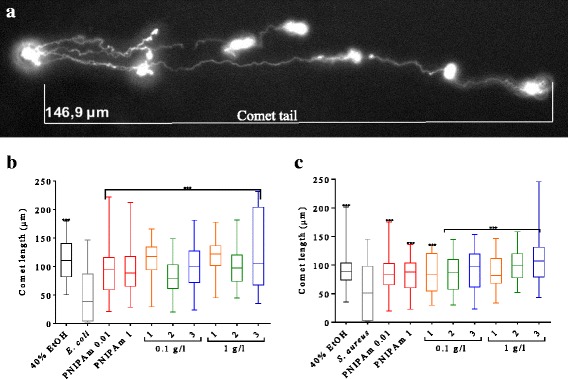
An example of an *Escherichia coli* comet tail*,* following treatment with Fe_3_O_4_-PNIPAAm-3 (**a**). Results of DNA strand breaks (length of comet tail) for *Escherichia coli* (**b**) and *Staphylococcus aureus* (**c**) incubated for 30 min with 40% EtOH (positive control), without nanocomposites (negative control), PNIPAAm (0.1 and 1 g/l), and (1) Fe_3_O_4_-PNIPAAm-1, (2) Fe_3_O_4_-PNIPAAm-2 and (3) Fe_3_O_4_-PNIPAAm-3 (error bars represent SD for comet length of 50 cells). Significance level ***P < 0.001

Following a short exposure of 30 min, DNA strand breaks were determined for both *E. coli* and *S. aureus* in cells treated with the Fe_3_O_4_-PNIPAAm nanocomposites, 40% EtOH (positive control) and untreated cells (negative control). All Fe_3_O_4_-PNIPAAm nanocomposites showed a similarly significant effect (*P* < 0.001) on mean *E. coli* and *S. aureus* comet tail length at all concentrations (Fig. 4), compared with control cells incubated without nanocomposites.

### Fe_3_O_4_-PNIPAAm Nanocomposite Antibacterial Effect

**Fig. 5:**
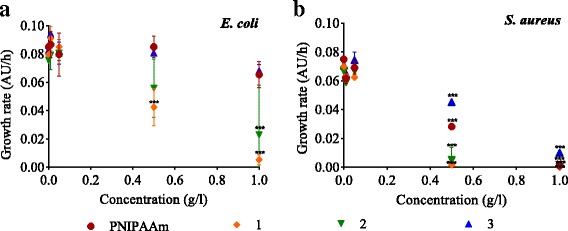
Relative growth rate of *Escherichia coli* (**a**) and *Staphylococcus aureus* (**b**) after 6-h incubation in different concentrations (0.01, 0.05, 0.5 and 1 g/l) of PNIPAAm (red circles), Fe_3_O_4_-PNIPAAm-1 (orange diamonds [1]), Fe_3_O_4_-PNIPAAm-2 (green triangles [2]) and Fe_3_O_4_-PNIPAAm-3 (blue triangles [3]). The error bars show SD calculated from *n* = 3. Significance level ***P < 0.001

Growth rates indicated that Gram-positive *S. aureus* was less resistant than Gram-negative *E. coli* to all nanocomposites after 6 h exposure. Fe_3_O_4_-PNIPAAm-1 and Fe_3_O_4_-PNIPAAm-2 both strongly inhibited bacterial growth, compared with PNIPAAm and Fe_3_O_4_-PNIPAAm-3, with *E. coli* growth rate significantly reduced from 0.08 to 0.028 (*P* < 0.001) with Fe_3_O_4_-PNIPAAm-2 and 0.005 (*P* < 0.001) with Fe_3_O_4_-PNIPAAm-1 (1 g/l). No effect was observed on *E. coli* growth rate by either PNIPAAm or Fe_3_O_4_-PNIPAAm-3 (Fig. 5a). In comparison, the growth rate of *S. aureus* was affected by all Fe_3_O_4_-PNIPAAm nanocomposites and by the PNIPAAm nanoparticles. At lower concentrations (0.01 g/l and 0.05 g/l), growth rate was only slightly reduced from 0.07 to 0.06 (*P* < 0.05). At higher concentrations (0.5 and 1 g/l), however, there was a significant reduction from 0.07 to 0.001 with PNIPAAm, 0.0 with Fe_3_O_4_-PNIPAAm-1, 0.01 with Fe_3_O_4_-PNIPAAm-2 and 0.009 with Fe_3_O_4_-PNIPAAm-3 (all *P* < 0.001; Fig. 5b). In addition, the EC10 for all Fe_3_O_4_-PNIPAAm nanocomposites and the PNIPAAm nanoparticle control was lower for *S. aureus* than that for *E. coli* (Table 1).

**Table 1:** The effective concentration of PNIPAAm, Fe_3_O_4_-PNIPAAm-1, Fe_3_O_4_-PNIPAAm-2 and Fe_3_O_4_-PNIPAAm-3 nanoparticles (g/l) at 10% growth inhibition (EC10) determined for Gram-negative *Escherichia coli* and Gram-positive *Staphylococcus aureus*

	PNIPAAm	Fe_3_O_4_- PNIPAAm-1	Fe_3_O_4_-PNIPAAm-2	Fe_3_O_4_-PNIPAAm-3
*E. coli*	0.43	0.11	0.14	0.67
*S. aureus*	0.10	0.05	0.04	0.06

**Fig. 6:**
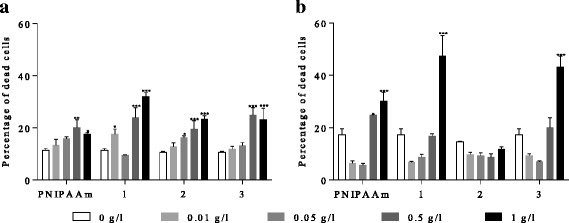
Percentage of dead *Escherichia coli* (**a**) and *Staphylococcus aureus* (**b**) cells after 24-h exposure to PNIPAAm and (1) Fe_3_O_4_-PNIPAAm-1, (2) Fe_3_O_4_-PNIPAAm-2 and (3) Fe_3_O_4_-PNIPAAm-3. Error bars show SD of *n* = 3. Significance levels *P < 0.05, **P < 0.005, ***P < 0.001

The percentage of dead *E. coli* cells increased with increasing concentration of Fe_3_O_4_-PNIPAAm nanocomposite after 24 h. PNIPAAm (0.5 and 1 g/l), for example, caused a significant increase in *E. coli* dead cells (20%) compared to cultures without Fe_3_O_4_-PNIPAAm nanocomposite (12%). Fe_3_O_4_-PNIPAAm-1 (0.5 g/l) resulted in up to 28% of dead *E. coli* cells and 32% at 1 g/l (*P* < 0.001). The effect of Fe_3_O_4_-PNIPAAm-2 was lower than that of Fe_3_O_4_-PNIPAAm-1 and Fe_3_O_4_-PNIPAAm-3, with the percentage of dead cells increasing from 13 to 25% when exposed to concentrations of 0.01 and 1 g/l, respectively (*P* < 0.001). At both 0.5 and 1 g/l, Fe_3_O_4_-PNIPAAm-3 resulted in around 25% dead cells (*P* < 0.001; Fig. 6a). The percentage of dead *S. aureus* cells was only significantly affected by 1 g/l Fe_3_O_4_-PNIPAAm-1 and Fe_3_O_4_-PNIPAAm-3, with dead cells reaching up to 50 and 48%, respectively (*P* < 0.001). The control without nanocomposites contained approximately 18% of dead cells while in lower concentrations of PNIPAAm, Fe_3_O_4_-PNIPAAm-1 and Fe_3_O_4_-PNIPAAm-3, the proportion of dead cells was even lower. PNIPAAm at concentrations of 0.5 and 1 g/l resulted in 25 and 30% (*P* < 0.005) dead cells, respectively. Fe_3_O_4_-PNIPAAm-2 had no effect on *S. aureus* cultures (Fig. 6b).

**Fig. 7:**
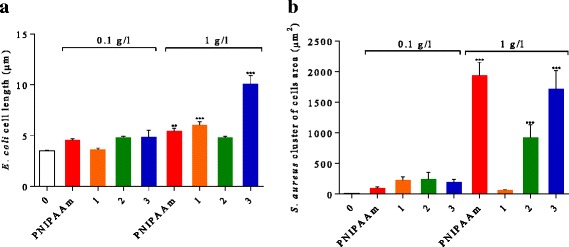
Length of *Escherichia coli* cells (**a**) and area of cluster of *Staphylococcus aureus* cells (**b**) after 24-h incubation with PNIPAAm and (1) Fe_3_O_4_-PNIPAAm-1, (2) Fe_3_O_4_-PNIPAAm-2 and (3) Fe_3_O_4_-PNIPAAm-3. The error bars show SD determined from *n* = 50. Significance levels **P < 0.05 and ***P < 0.001

There was no difference in average *E. coli* cell length (5 μm) and average *S. aureus* cell cluster area (200 μm^2^) for any nanocomposite or the PNIPAAm control at lowest concentrations (0.1 g/l; Fig. 7). At higher concentrations, *E. coli* length did not change in the presence of Fe_3_O_4_-PNIPAAm-2, nor did *S. aureus* cell group area in the presence of Fe_3_O_4_-PNIPAAm-1. However, *E. coli* length was significantly increased in the presence of 1 g/l of PNIPAAm (5.4 μm, *P* < 0.005), Fe_3_O_4_-PNIPAAm-1 (6 μm, *P* < 0.001) and Fe_3_O_4_-PNIPAAm-3 (10 μm, *P* < 0.001) (Fig. 7a), while *S. aureus* formed larger clusters when exposed to PNIPAAm (1937 μm^2^, *P* < 0.001), Fe_3_O_4_-PNIPAAm-2 (924 μm^2^, *P* < 0.001) and Fe_3_O_4_-PNIPAAm-3 (1722 μm^2^, *P* < 0.001) (Fig. 7b).

## Discussion

Both the method of synthesis and the means by which magnetic nanoparticles were added to the polymer matrix had a clear effect on the intrinsic physical-chemical properties of the magnetic Fe_3_O_4_-PNIPAAm nanocomposites. Stepwise synthesis had a strong impact on nanocomposite properties, resulting in changes to particle shape, size distribution, size and surface chemistry, along with subsequent changes in magnetic properties [19, 20]. Emulsion polymerisation (Fe_3_O_4_-PNIPAAm-1), an easy and precise method, produced the stable nanocomposites with narrow particle size distribution and lowest aggregation tendency, qualities particularly important in biomedical applications [17]. Produced as a result of both steric and coulombic repulsion, the particle dimensions were sufficiently small that precipitation was avoided [21]. The least effective method was physical addition (Fe_3_O_4_-PNIPAAm-3). Not only was it produced via three distinct steps, and hence took longer to prepare, the resulting nanocomposite showed higher aggregation than either of the other two production methods. Moreover, our results indicated that Fe_3_O_4_-PNIPAAm-3 produced in this way may have contained undesirable PNIPAAm and Fe_3_O_4_ nanoparticle residuals.

Polymers can become attached to magnetic nanoparticles by either physical (noncovalent) or covalent bonds, with the resulting hybrid material displaying specific properties depending on the synthetic route taken. Significant re-suspension of magnetic nanoparticles takes place when preparation proceeds in the solvent in which hybrid nanoparticle formation occurs, whereupon aggregation and segregation may become a problem. In this case, in situ formation of magnetic nanoparticles may be a better alternative in many cases. In addition, if the surfactant concentration is too low, coalescence will change the size of the droplets, whereas micelles can form if the concentration is too high, leading to micellar nucleation. In this respect, it is important that the surfactant concentration is chosen carefully based on precise characterisation of surface properties and extent of particle modification, in order to ensure the inorganic particle surface is compatible with the polymer matrix.

In order to evaluate the magnetic properties of Fe_3_O_4_-PNIPAAm nanocomposites, it is important to know the content of MNPs in the nanocomposite. TGA was employed to quantify amount of MNP and to investigate thermal stability of Fe_3_O_4_-PNIPAAm nanocomposites compared with PNIPAAm nanoparticles alone. All three Fe_3_O_4_-PNIPAAm nanocomposites displayed higher thermal stability than PNIPAAm nanoparticles, presumably due to the presence of Fe_3_O_4_ particles in the matrix (Fig. 2). Higher residues in magnetic nanocomposites could be attributed to the presence of inorganic Fe_3_O_4_ compounds in the samples, which were sustained even at higher temperatures.

Fe_3_O_4_-PNIPAAm-1 showed highest thermal nanocomposite stability, along with the lowest weight lost. Up to 200 °C, the main source of weight loss was through loss of water and physical adsorption of the polymer layer [22]; above 200 °C, however, losses were mainly due to decomposition of the chemical layer bonding the PNIPAAm. The sample residue, which became stable above 400 °C, represented 87% of the original weight, which corresponds with the amount of magnetic nanoparticles in the nanocomposite. One aim of this preparation process was to produce a nanocomposite with magnetic properties preventing aggregation and enabling it to re-disperse rapidly as soon as the magnetic field is turned off. Such properties would allow its use in a range of different fields, including hyperthermic treatment of tumours, as contrasting agents in magnetic resonance imaging, in tissue repair, biomedical device coating, immunoassay, cell separation and biomagnetic separation of biomolecules [18, 23–26]. We tested our nanomaterials through magnetisation saturation, which assesses the maximum possible magnetisation of the substance beyond which no further change takes place despite an increase in the magnetic field. Our results showed Fe_3_O_4_-PNIPAAm-2 to have the highest magnetisation saturation level of the three nanocomposites tested. Our values were lower (53.7 emu/g) than those previously reported for uncoated Fe_3_O_4_ nanoparticles (92 emu/g) [27], however, presumably due to surface order/disorder interactions in the magnetic spin moment and an increase in nanocomposite weight and volume due to the presence of the PNIPAAm polymer layer.

Of special interest as regards biomedical application is the behaviour of polymer-water solutions stable below a LCST [28]. After heating the prepared Fe_3_O_4_-PNIPAAm nanomaterials above the transition temperature, a coil-to-globule transition occurred, followed by inter-molecular association. All three Fe_3_O_4_-PNIPAAm nanomaterials displayed very similar behaviour, with all shrinking as temperature increased. PNIPAAm is widely used as a thermoresponsive polymer due to the proximity of its LCST (~ 30–32 °C) to physiological temperature. Furthermore, the thermo-responsibility of PNIPAAm has proved useful for drug release in vivo [28]. Nanoscale magnetic hydrogels based on PNIPAAm have now been developed for theranostic application, with those embedded with low concentrations of Fe_3_O_4_ magnetic nanostructures resulting in an LCST of ~ 40 °C, making Fe_3_O_4_-PNIPAAm of especial interest for controlled drug release application [29].

SEM nanoparticle histograms displayed a broader size distribution than those using DLS (Fig. 1). Interpretation of DLS data involves the interplay of multiple parameters, however, including the size, concentration, shape, polydispersity and surface properties of the particles. Measurement of the hydrodynamic size of thermoresponsive samples in relation to temperature is a common method of characterising LCST behaviour, with nanoparticles shrinking as temperatures increase, soluble polymers precipitating and particle size increasing. As expected, PNIPAAm had a lower hydrodynamic size than the Fe_3_O_4_-PNIPAAm nanocomposites. Of the nanocomposites, Fe_3_O_4_-PNIPAAm-3 displayed the lowest hydrodynamic size and a narrow size distribution. Variability in hydrodynamic size is likely to be due to the presence of Fe_3_O_4_ nanoparticles in the PNIPAM matrix, which increases both the particle dimension and aggregation in water (Fig. 3) [8].

All Fe_3_O_4_-PNIPAAm nanocomposites displayed antimicrobial properties (Table 2), with both Gram-negative and Gram-positive bacteria negatively affecting *E. coli* growth rate in the order Fe_3_O_4_-PNIPAAm-1 > Fe_3_O_4_-PNIPAAm-2 > Fe_3_O_4_-PNIPAAm-3 = PNIPAAm and *S. aureus* growth rate as Fe_3_O_4_-PNIPAAm-1 > Fe_3_O_4_-PNIPAAm-2 > Fe_3_O_4_-PNIPAAm-3 > PNIPAAm. Similarly, the antibacterial properties desired for medical applications such as biomedical device coatings and wound dressing materials have been confirmed for a number of new PNIPAAm composites, including ZnO-PNIPAAm, Ag-PNIPAAm and chitosan-PNIPAAm [23–26].

**Table 2:** Summary of the antimicrobial effect of PNIPAAm and three nanocomposites on *Escherichia coli* and *Staphylococcus aureus*

Endpoint Strain	PNIPAAm	Fe_3_O_4_-PNIPAAm-1	Fe_3_O_4_-PNIPAAm-2	Fe_3_O_4_-PNIPAAm-3
Growth rate				
*E. coli*	0	– –	–	0
*S. aureus*	–	– –	– –	–
Viability				
*E. coli*	– –	– –	– –	– –
*S. aureus*	– –	– –	0	– –
DNA damage				
*E. coli*	–	– –	– –	– –
*S. aureus*	–	– –	– –	– –

*0* no effect, − significant negative effect, − – strong negative effect

In comparison with the modified Fe_3_O_4_ nanomaterials described in our earlier studies, the PNIPAAm-1, PNIPAAm-2 and PNIPAAm-3 nanocomposites all showed a stronger effect on both *E. coli* and *S. aureus*, with *S. aureus* EC10 growth inhibition ranging from 0.04 to 0.06 g/l for the three nanomaterials, while modified APTS-, PEG- and TEOS-MNPs ranged between 0.1 and 0.25 g/l [17], and polymer-coated Fe_3_O_4_ (PEI-mC-, PEI- and OA-MNPs) had a value of 0.15 g/l [18]. Inhibition of bacterial growth could have been caused by several factors, including cell membrane damage, oxidative stress and cell elongation, resulting in the production of lethal cells. The cells could, on the other hand, survive such unfavourable conditions by employing repair enzymes, antioxidants and/or transient growth arrest. This could partly explain the phenomenon that in lower concentrations (0.01 and 0.05 g/l) of PNIPAAm, Fe_3_O_4_-PNIPAAm-1 and Fe_3_O_4_-PNIPAAm-3, the proportion of dead cells of *S. aureus* was lower after 24-h incubation than in control where no such factor inducing mobilisation of the defence/repair system was present. Higher concentrations of PNIPAAm and nanocomposites caused indeed significant increase in dead cells of *E. coli* and *S. aureus* corresponding well with significant decrease in growth rate of the cell cultures.

**Fig. 8:**
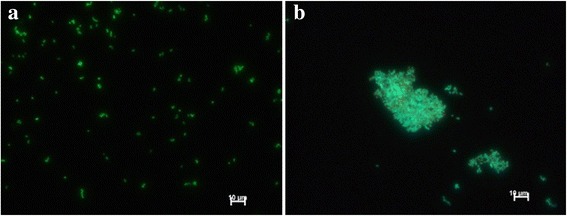
*S. aureus* cell culture without nanocomposites (**a**) and the cells embedded in biofilm after incubation with nanocomposites for 24 h (**b**). The scale bar is 10 μm

Exposure to 1 g/l of the nanocomposite resulted in changes to bacterial cell morphology, with greatest change to *E. coli* cell length caused by Fe_3_O_4_-PNIPAAm-3 > Fe_3_O_4_-PNIPAAm-1 > PNIPAAm > Fe_3_O_4_-PNIPAAm-2, and Fe_3_O_4_-PNIPAAm-3 > Fe_3_O_4_-PNIPAAm-2 > PNIPAAm > Fe_3_O_4_-PNIPAAm-1 for *S. aureus* clustering. This effect was also observed previously when the same bacteria were exposed to different functional magnetic nanoparticles [17]. Elongation of *E. coli* cells in the presence of nanocomposites is indicative of transient growth arrest and is evidence of an adaptive response to oxidative stress or DNA damage [30]. In the case of *S. aureus*, which is a biofilm formation species, the cells became embedded over a larger area than the nanocomposite-free control when exposed to PNIPAAm, Fe_3_O_4_-PNIPAAm-2 and Fe_3_O_4_-PNIPAAm-3 (Fig. 8). No *S. aureus* biofilm was produced when in contact with Fe_3_O_4_-PNIPAAm-1, possibly due to its stronger antibacterial properties. *S. aureus* usually produces a biofilm in harsh environments to protect the cells [31]; however, this could also have an adverse effect on the bacteria as nanocomposites can integrate through the biofilm and harm the cells, as has already been described for *Pseudomonas* sp. [32].

Iron could lead to DNA damage in bacterial cells as described in previous reviews [33, 34]; hence, we attempted to test whether our MNPs caused DNA damage to bacteria. The presence of Fe_3_O_4_-PNIPAAm nanocomposites at both low and high concentrations (0.01 or 1 g/l) caused significant damage to *E. coli* and *S. aureus* DNA, even after short exposures (30 min). To the best of our knowledge, this is the first acute genotoxicity study of magnetic composites on bacteria; as a result, we cannot compare our results with those of other authors directly. Previous studies have shown no genotoxicity attributable to PNIPAAm nanoparticles, however, and no decrease in cell viability when tested against two kinds of mammalian cell at nanoparticle concentrations of up to 800 mg/l [30]. On the other hand, previous genotoxicity studies on MNPs (γ-Fe_3_O_4_) have shown a negative effect on human fibroblast cells at 100 mg/l [35]. Studies performed with mammalian cell lines, however, cannot be directly compared to studies done with bacterial cells, due to significant differences in eukaryotic and prokaryotic cells.

## Conclusions

Magnetic poly(N-isopropyl-acrylamide) nanocomposites were prepared through emulsion polymerisation (Fe_3_O_4_-PNIPAAm-1), in situ precipitation (Fe_3_O_4_-PNIPAAm-2) and physical addition (Fe_3_O_4_-PNIPAAm-3). Both Fe_3_O_4_-PNIPAAm-1 and Fe_3_O_4_-PNIPAAm-2 showed higher values for surface charge and thermal stability, indicating a stable colloidal system. At room temperature, Fe_3_O_4_-PNIPAAm-3 displayed highest magnetisation saturation. Presence of Fe_3_O_4_-PNIPAAm nanocomposites at both low and high concentrations caused significant damage to both *E. coli* and *S. aureus* DNA, even after short exposure, and led to a decrease in cell viability. Overall, we suggest that Fe_3_O_4_-PNIPAAm-1, prepared through emulsion polymerisation, is the most appropriate method for producing a magnetic nanocomposite with high antimicrobial activity towards Gram-negative *E. coli* and Gram-positive *S. aureus*.

## Abbreviations

Fe_3_O_4_-PNIPAAm: Magnetic poly(N-isopropylacrylamide)
MNPs: Magnetite nanoparticles
